# Wunderlich Syndrome Managed with Angiomyolipoma Embolization – Renal Artery Anatomic Variant Augmenting Safe and Nephron-Sparing Intervention

**DOI:** 10.15388/Amed.2024.31.1.3

**Published:** 2024-02-27

**Authors:** Tara Prasad Tripathy, Alamelu Alagappan, Ranjan Patel, Srikant Kumar Behera, Panda Sandip Kumar, Suprava Naik

**Affiliations:** 1 Department of Radiodiagnosis, AIIMS, Bhubaneswar, India; 2 Department of Medicine, AIIMS, Bhubaneswar, India; 3 Department of Nephrology, AIIMS, Bhubaneswar, India

**Keywords:** Wunderlich syndrome, embolization, angiomyolipoma, Wunderlicho sindromas, embolizacija, angiomiolipoma

## Abstract

**Background:**

Wunderlich syndrome is an uncommon entity characterized by spontaneous, nontraumatic renal bleeding into the subcapsular and perirenal regions. The most frequent benign tumor, angiomyolipoma, is the most common cause of Wunderlich syndrome

**Case presentation:**

We report a case of Wunderlich syndrome in angiomyolipoma. Intratumoral pseudoaneurysm arising from feeders of an accessory renal artery supplying the lower pole of the kidney was selectively embolized. Rarely does a sporadic renal angiomyolipoma develop a giant pseudoaneurysm

**Conclusion:**

Transarterial embolization is imperative to control the bleeding or as a preventative measure to reduce the risk of intralesional pseudoaneurysm rupture. When vascular interventional facilities are unavailable, surgery may be necessary

## Abstract (it)

**Kontekstas:**

Wunderlicho sindromas yra nedažnas reiškinys, jį apibūdina spontaniškas netrauminis inkstų kraujavimas į subkapsulinę ir perirenalinę erdvę, dažniausiai dėl gerybinio auglio – angiomiolipomos

**Atvejo pristatymas:**

Pateikiame Wunderlicho sindromo su angiomiolipoma ataskaitą. Auglio vidinė pseudoaneurizma, atsiradusi iš inkstų arterijos kanalų, tiekiančių kraują į apatinį inksto polių, buvo selektyviai embolizuota. Iš sporadinės inkstų angiomiolipomos išsivysčiusi didžiulė pseudoaneurizma yra retas reiškinys

**Išvada:**

Transarterinė embolizacija būtina kraujavimo kontrolei arba kaip prevencinė priemonė intralezinio pseudoaneurizmos plyšimo rizikai sumažinti. Kai neįmanoma pasitelkti vaskulinių intervencinių priemonių, chirurginė operacija gali būti neišvengiama

## Introduction

Angiomyolipoma (AML) is one of the most common benign renal tumors., with a prevalence of 0.2% to 0.6% and a strong female predilection [1]. In 80% of cases, AML appears as a sporadic or isolated entity. The remaining 20% of AMLs are associated with pulmonary lymphangioleiomyomatosis (LAM) or tuberous sclerosis complex (TSC) [2]. The majority (> 80%) of AMLs are now discovered inadvertently, which can be explained by the increased use of cross-sectional imaging and technological advancements in imaging [3,4]. Wunderlich syndrome (spontaneous perirenal hemorrhage) and retroperitoneal hemorrhage (< 15% of cases) are the most common causes of symptomatic presentation [5]. As a result, one-third of the individuals may present with shock [1]. Thus, the most significant clinical worry in a patient with an AML diagnosis is the possibility of a life-threatening hemorrhage.

Selective embolization of renal angiomyolipoma is a safe and minimally invasive technique with few complications. As opposed to renal resection, it spares the nephron. After embolization, the tumour’s size is significantly and permanently reduced.

We describe a rare instance of a 38-year-old woman without tuberous sclerosis who presented with left flank pain and tenderness for three days. Wunderlich syndrome with giant intratumoral pseudoaneurysm in angiomyolipoma diagnosed on Contrast Enhanced Computed Tomography (CECT). The patient underwent emergency selective renal angiography with embolization to halt the perirenal hemorrhage and avoid potential rebleeding.

## Case report

A 38-year-old lady presented to the ER with complaints of acute severe left-sided flank pain. Blood pressure at the time of admission was 90/60 mm Hg, PR 110 bpm. The rest of the vitals were normal. On examination, a vague mass with tenderness was noted in the left lumbar region. Laboratory reports revealed Hb 7.5 mg/dl and serum creatinine 1.1 mg/dl. The rest of the other laboratory parameters were unremarkable. Ultrasonography done outside revealed left perirenal hematoma.

**Figure 1:**
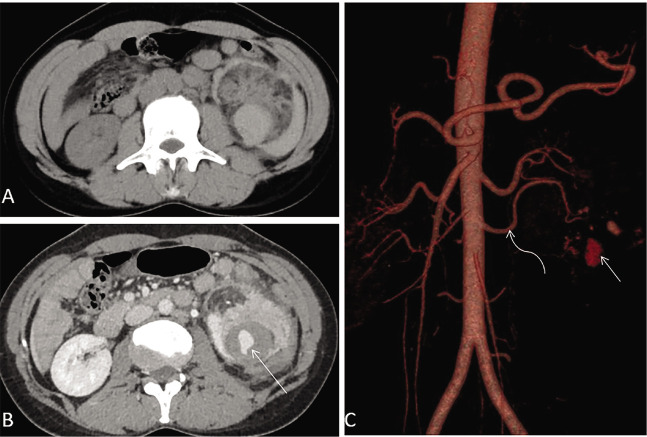
A) Noncontrast CT axial section showing heteroattenuating lesion in left kidney with intratumoral fat density and perinephric hematoma. B) CECT showing intratumoral ruptured pseudoaneurysm (straight arrow). C) Virtual 3D reconstruction showing left accessory renal artery supplying lower pole of left kidney (curved arrow).

CECT revealed a large (7.6x5.5x4.2 cm) well-defined fat-containing mass arising from the lower pole of the left kidney with a large ruptured intratumoral pseudoaneurysm. Extensive perirenal hematoma was seen. An unruptured aneurysm was also seen within the tumour. The angiographic anatomical evaluation revealed that the accessory left renal artery supplying the lower pole of the kidney provided the feeders to the AML (Figure 1). No features of tuberous sclerosis were present either clinically or radiologically.

**Figure 2:**
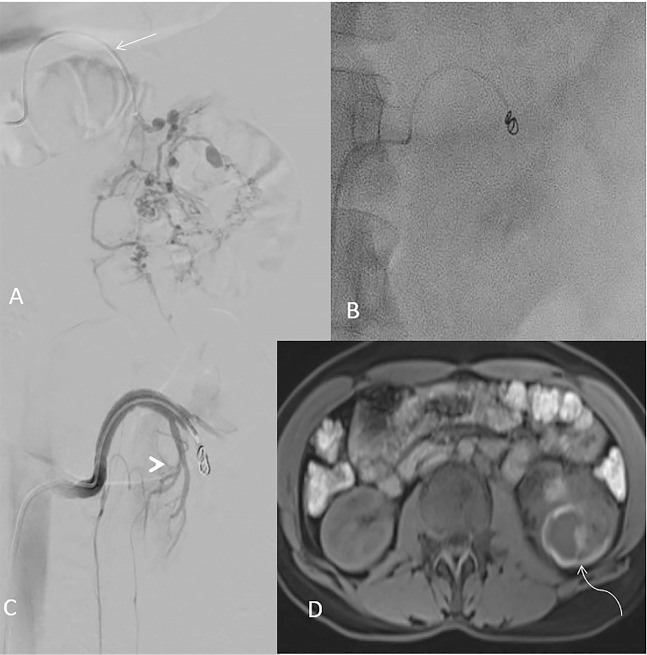
A) Digital subtraction angiography run from left accessory renal artery (straight arrow) showing large angiomyolipoma with multiple intrasac pseudoaneurysms embolized using PVA particles. B) Fluoroscopic image showing coiling of feeder artery. C) Post-coiling angiography showing superselective and complete embolization of angiomyolipoma. Supply to normal renal parenchyma preserved (arrow head). D) Post-embolization (at 3 months) MRI T1W imaging showing reduction in tumor size (curved arrow).

A final diagnosis of sporadic angiomyolipoma with Wunderlich syndrome was made, and after multi-disciplinary team discussion, decision for superselective embolization was taken. The patient was immediately shifted to the angiography suite for selective embolization. Vascular access through the right common femoral artery using the Seldinger technique. The left renal artery was selectively catheterized with a 5 Fr Cobra catheter and an angiographic run showed no supply to AML. The left lower lobar branch of the accessory renal artery supplying the lesion was cannulated. The artery feeding the AML was cannulated superselectively using a microcatheter (2.7Fr Progreat, Terumo), and an angiographic run revealed the large AML with multiple intratumoral aneurysms. PVA particles (300 to 500 microns) were used, followed by coiling of the feeder artery to AML using an 18-5-3 coil (Hilal, Cook). Post-coiling angiographic run was taken, showing complete embolization of angiomyolipoma and sparing of lower pole arterial supply to normal renal parenchyma (Figure 2).

The patient was shifted to the post-operative ward and managed with antibiotics, analgesics and antiemetics. The patient was discharged on second post-operative day and is on follow-up. Being a radiation free cross-sectional modality, MRI was done three months later, which revealed regression in the size (4.5x3x3 cm) of the angiomyolipoma with no recurrence of pain or bleeding episodes.

## Discussion

AML includes a heterogeneous group of neoplasms. Adipose skin, smooth muscle, and blood vessels are present in varied amounts in all forms, despite many of them having diverse pathological and radiological features and clinical behavior. Although some renal AMLs are asymptomatic, they tend to enlarge and can result in local symptoms. Common signs of renal AMLs include flank discomfort, palpable mass, blood in urine, fall in haemoglobin, and symptoms of a mass effect such as abdomen pain, visceral compression by the mass, and anorexia [6]. In our case, the patient complained of sudden onset left-sided flank pain. Flank mass and hypotension were elicited subsequently.

Potential life-threatening complications in angiomyolipoma include retroperitoneal hemorrhage, gross haematuria, and perirenal hemorrhage (Wunderlich syndrome), causing hemorrhagic shock. Wunderlich‘s syndrome, also known as „spontaneous nontraumatic retroperitoneal hemorrhage,“ is one of the most prevalent causes of mortality in people with angiomyolipoma and is typically limited to the renal subcapsular space or the perinephric region.

The severity and duration of the bleeding determine the prognosis. The typical manifestation of Wunderlich‘s syndrome is known as Lenk‘s triad, which comprises flank or abdominal pain, a palpable painful mass, and extensive haematuria [5]. The patient in this case study presented to emergency with a hemorrhagic shock due to a life-threatening perirenal hemorrhage (Wunderlich syndrome).

Renal AMLs feature abnormal blood arteries that are rigid, tortuous, and prone to aneurysm formation and rupture because they lack an internal elastic lamina and smooth muscle that has been replaced by fibrous tissue [3]. During their clinical course, renal AMLs have been proven to have a high chance of aneurysmal rupture.

Possible treatments include the mammalian target of rapamycin (mTOR) inhibitor therapy, nephron-sparing surgery, partial/total nephrectomy, cryo- and radio-frequency ablation, and selective arterial embolization (SAE) [4].

An effective RML treatment is superselective arterial embolization (SAE). Most patients with AMLs can have their bleeding controlled with this minimally invasive procedure. Moreover, the remaining kidney parenchyma retains its functionality once the bleeding vessel has been successfully superselectively embolized. As a result, it was regarded as the preferred method of managing symptomatic AMLs in a few trials [7].

In the present case, superselective embolization was successfully performed using microparticles and coils. Many authors claimed to have 100% success rates [8, 9].

In their literature review, Murray et al. reported that the side effects of embolization include allergic reactions to contrast (in 0.6% cases), nontargeted embolization in 2.3% cases, respiratory issues (~2%), retroperitoneal hemorrhage (~1%), and hematoma at the site of the punctured femoral artery. These issues, meanwhile, do not pose a life-threatening complication [10].

Radeleff et al. in their study showed that transarterial embolization could be used more frequently to treat large, asymptomatic tumors (>4 cm) and small symptomatic tumors (<4cm) with successfully managing symptoms in 80–93% of cases, thereby stopping bleeding in the majority of renal AML patients. A significant reduction in tumor size was also shown [11].

## Conclusion

If technically possible, patients with life-threatening hemorrhage in angiomyolipomas should first be treated with selective arterial embolization since it is nephron-sparing. All sporadic AMLs should undergo annual clinical and imaging evaluation.
